# Employing Machine Learning-Based QSAR for Targeting Zika Virus NS3 Protease: Molecular Insights and Inhibitor Discovery

**DOI:** 10.3390/ph17081067

**Published:** 2024-08-15

**Authors:** Hisham N. Altayb, Hanan Ali Alatawi

**Affiliations:** 1 Department of Biochemistry, Faculty of Science, King Abdulaziz University, Jeddah 21589, Saudi Arabia; 2 Department of Biological Sciences, University Collage of Haqel, University of Tabuk, Tabuk 71491, Saudi Arabia; halatwi@ut.edu.sa

**Keywords:** bioactive phytochemicals, antiviral agents, Flavivirus, drug discovery, in silico

## Abstract

Zika virus infection is a mosquito-borne viral disease that has become a global health concern recently. Zika virus belongs to the Flavivirus genus and is primarily transmitted by Aedes mosquitoes. Prevention of Zika virus infection involves avoiding mosquito bites by using repellent, wearing protective clothing, and staying in screened areas, especially for pregnant women. Treatment focuses on managing symptoms with rest, fluids, and acetaminophen, with close monitoring for pregnant women. Currently, there is no specific antiviral treatment or vaccine for the Zika virus, highlighting the importance of prevention strategies to control its spread. Therefore, in this study, the Zika virus non-structural protein NS3 was targeted to inhibit Zika infection by identifying the novel inhibitor through an in silico approach. Here, 2864 natural compounds were screened using a machine learning-based QSAR model, and later docking was performed to select the potential target. Subsequently, Tanimoto similarity and clustering were performed to obtain the potential target. The three most potential compounds were obtained: (a) 5297, (b) 432449, and (c) 85137543. The protein–ligand complex’s stability and flexibility were then investigated by dynamic modelling. The 300 ns simulation showed that 5297 exhibited the steadiest deviation and constant creation of hydrogen bonds. Compared to the other compounds, 5297 demonstrated a superior binding free energy (ΔG = −20.81 kcal/mol) with the protein when the MM/GBSA technique was used. The study determined that 5297 showed significant therapeutic potential and justifies further experimental investigation as a possible inhibitor of the NS2B-NS3 protease target implicated in Zika virus infection.

## 1. Introduction

The Zika virus (ZIKV) is a mosquito-borne Flavivirus [1] that has emerged as a significant health concern due to its ability to cause severe neurological complications, such as congenital abnormalities in newborns and Guillain–Barré syndrome in adults [2]. It was first identified in Uganda in 1947 in monkeys through a network that monitored yellow fever. It was later identified in humans in 1952 in Uganda and the United Republic of Tanzania [3]. The Zika virus is primarily transmitted by Aedes mosquitoes, such as Aedes aegypti and Aedes albopictus. These mosquitoes are also vectors for other viruses like dengue, chikungunya, and yellow fever [3,4]. The Zika virus has a single-stranded, positive-sense RNA genome of approximately 10.7 kilobases in length. The genome consists of a single open reading frame (ORF) flanked by untranslated regions (UTRs) at the 5′ and 3′ ends [5]. The single ORF encodes a polyprotein that is co- and post-translationally processed into three structural proteins and seven non-structural proteins. The non-structural proteins are essential for viral replication, polyprotein processing, and modulation of host cellular processes [6]. Compounds that specifically target the RNA-dependent RNA polymerase or methyltransferase activities of NS5 (non-structural protein 5) have the ability to impede the process of viral RNA synthesis and capping. Compounds that hinder the protease or helicase functions of NS3 (non-structural protein 3) or interfere with the creation of the NS2B-NS3 (non-structural protein 2B and non-structural protein 3 protease) protease complex have the ability to impede polyprotein processing and the reproduction of the virus. In addition, specifically targeting the functions of NS1 (non-structural protein 1) can help restore the host’s immune response and disrupt viral replication [7]. Various studies are investigating small-molecule inhibitors, peptide-based inhibitors, and repurposed drug candidates to potentially delay the Zika virus infection by targeting its non-structural proteins.

Despite ongoing studies, there are currently no approved antiviral therapies or vaccines available for the treatment or prevention of Zika virus infections [8]. A potential approach for developing antiviral drugs for the prevention of ZIKV involves focusing on the non-structural protein 3 (NS3). NS3 is a viral enzyme that plays a crucial role in the replication cycle of the virus. Specifically, the NS3 serine protease domain is responsible for cleaving the viral polyprotein, a crucial step in the maturation and assembly of new viral particles [9,10]. Inhibiting the NS3 serine protease could potentially disrupt the viral replication process and halt the progression of Zika virus infection [11]. The study by Rehman et al. (2022) showed that in silico drug exploration identified potent inhibitors of Zika NS2B-NS3 protease through e-pharmacophore-based virtual screening and MD simulations [12]. Another study by Giarolla et al. (2022) focuses on computational docking simulations of active peptides against the NS2B-NS3 protease of Zika virus, analysing quantum chemical properties to enhance inhibitory action [13]. Therefore, in this study, an integrative computational approach combining quantitative structure–activity relationship (QSAR) modelling, molecular docking, and molecular dynamics (MD) simulations has been employed to identify potential inhibitors of the ZIKV NS3 serine protease. This comprehensive approach utilises diverse computational approaches to provide an extensive assessment of possible inhibitor candidates.

## 2. Results and Discussion

### 2.1. Protein Structure and Compound Library

**Figure 1:**
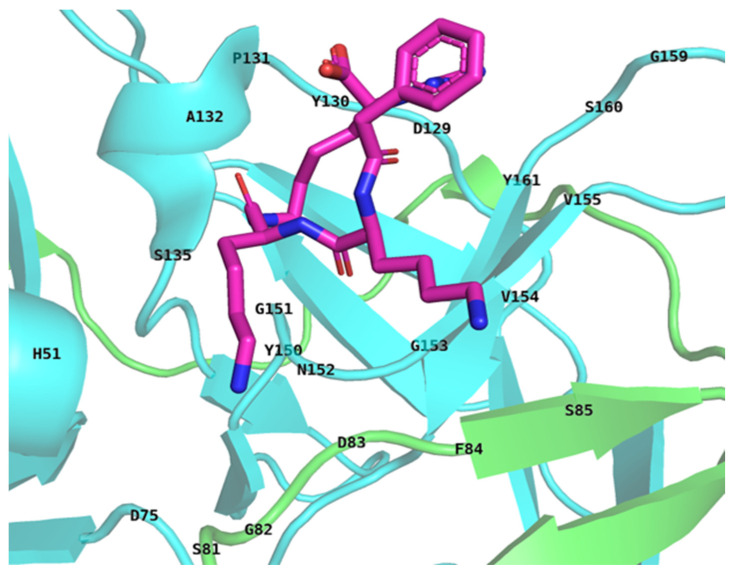
The binding residues have been identified using the PyMOL tool, version 2.6; the binding residues are those located within a 6 Å radius around the ligand.

The crystal structure of NS2B_NS3 protease was collected from the PDB, and chain a (serine protease subunit NS2B) and chain b (serine protease NS3) were both used in the input. The binding site residues of the structure were identified using PYMOL. The binding residues are those located within a 6 Å radius around the ligand, as shown in Figure 1. Furthermore, the NS2B_NS3 protease was co-crystallised with a ligand (MI-2113), and residues within a 6 Å distance from the native ligand (MI-2113) were used to form the grid box. This compound, MI-2113, was used as a ligand in further studies. While a distance of 6 Angstroms (Å) is considered relatively large, it offers an advantage during the docking and simulation processes. Although strong interactions typically occur at distances shorter than 6 Å, this larger distance allows for wider exploration of the ligand molecule’s conformational space. Consequently, the ligand has a greater opportunity to sample various orientations and positions, increasing the chances of attaining its most stable and preferred binding pose within the target protein’s active site. Moreover, the natural product library from the Selleckchem database was selected for screening against the NS2B_NS3 protease. In total, 2864 natural compounds were obtained from the natural product library.

### 2.2. Machine Learning-Based Compound Screening

Quantitative structure–activity relationship (QSAR) machine learning models are very effective computational methods used to screen and predict the biological activities or attributes of chemical compounds. These models utilise the ideas of QSAR, which establish mathematical correlations between the structural features or descriptors of molecules and their observable activities or qualities [14]. Machine learning-based QSAR models are commonly used in drug discovery and chemical safety assessment. Machine learning QSAR algorithms can predict the biological activity of large chemical libraries, pinpointing compounds that may have activity against a particular target [15]. Here, in this study, the ”Targets” part of the ChEMBL database was searched using the term “ZIKA”, leading to the identification of an individual target ID (CHEMBL4296612). The target ID had 262 compounds with activity (EC50), while only 158 compounds were retained after removing blanks and duplicates. The EC50 value, commonly found in pharmacology and toxicology, is a term that represents the concentration of a substance required to produce 50% of its maximal effect. Similarly, to predict EC50 values using machine learning-based QSAR models, one must compile a dataset containing details on chemical structures and their respective experimental EC50 values. Subsequently, molecular descriptors (morgan fingerprints) should be computed to depict the chemical structures. A machine learning model can then be developed using these descriptors and EC50 values, followed by model validation through methods such as cross-validation. Lastly, new chemical structures can be added to the model to predict their EC50 values. Afterwards, 158 compounds were normalised by taking the log10 values and converting them to nM (nanomolar). Moreover, 70% of the compounds were designated for training the models, whereas 30% were allocated for the test set. In this study, using 110 compounds was sufficient for building a robust and predictive model, and several studies in the field have successfully employed similar dataset sizes. Many studies have demonstrated successful QSAR model development with comparable datasets; a previous study on a training set of 30 compounds was conducted by Darnag et al. [16]. Another study also used a similar dataset, Shoombuatong et al. [17]. The log scale standardises the scale of all compounds, ensuring uniformity throughout model training and reducing bias.

Furthermore, the R^2^ value was calculated for the validation of the models; the R^2^ indicates the degree of correlation between predicted and observed data, which may be used to evaluate the model’s performance. Here, the evaluation of all the models showed promising outcomes, with the highest coefficient of determination (R^2^) value attained being 0.79, as listed in Table 1. This suggests that among the models considered, the random forest (RF) model exhibited the strongest predictive performance, as indicated by its superior R^2^ value. Later, the trained model was applied to conduct QSAR analysis on 2864 natural compounds obtained from the Selleckchem natural product library and the control group to assess their activity levels. Compound EC50 was predicted using antilog10, and as an outcome, 221 compounds showed better activity compared to the control group, and after the removal of duplicates and blanks, 205 compounds were retained and used for further analysis.

### 2.3. Molecular Docking

In the present study, the protein 3D crystal structure (PDB: 7ZMI) was loaded in the Autodock tool with the grid box information and docked against the 205 screened compounds of the NS2B_NS3 protease. Later, the binding energy of all 205 compounds was normalised as shown in Table S1, and based on the normalised score, 28 compounds were selected that showed better binding energy than the control (MI-2113). These 28 compounds showed binding energy in the range of −7 kcal/mol to −9 kcal/mol. Further, the normalised score was also in the range −6.8 kcal to −8.6 kcal/mol.

### 2.4. Tanimoto Similarity and K-Mean Clustering

**Figure 2:**
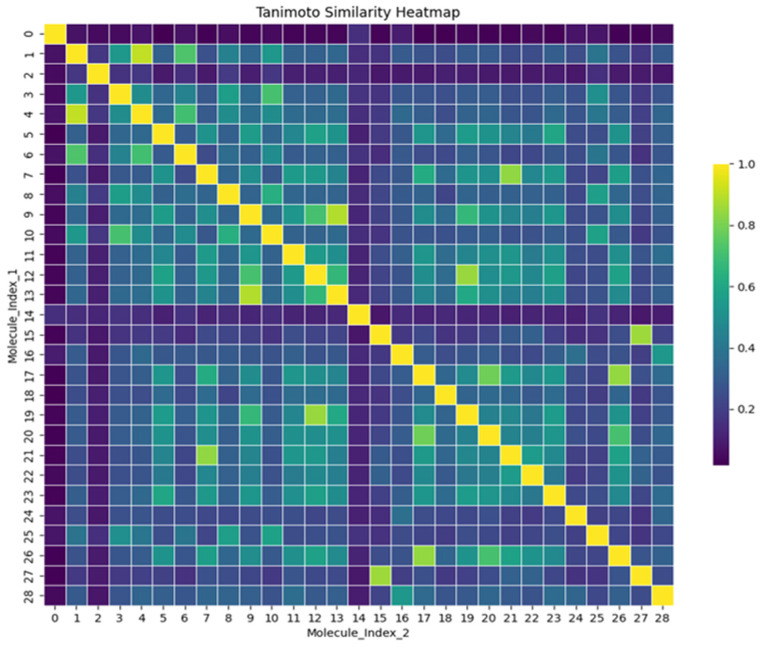
Tanimoto similarity heatmap. The matrix is symmetric, with the diagonal representing the self-similarity of each compound (a value of 1.0). The colour scale indicates the degree of similarity between pairs of compounds, with yellow representing high similarity and purple representing low similarity.

**Figure 3:**
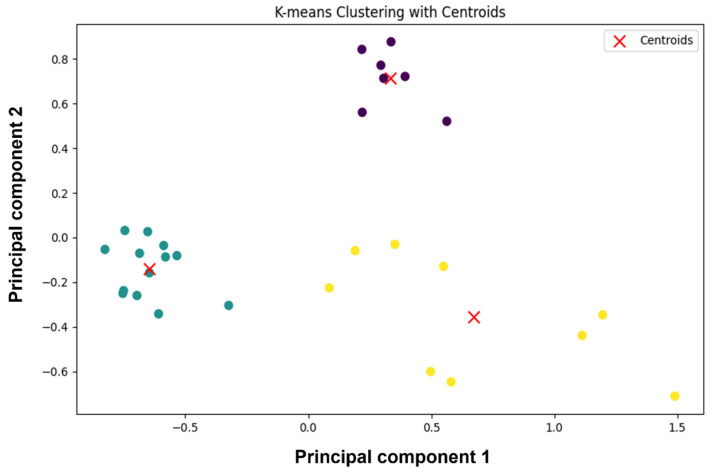
K-means clustering visualisation displays data partitioned into three distinct groups, each represented by a unique colour, with red ‘X’ marks identifying the centroids of each cluster on a plot of the first two principal components.

The 28 compounds were looked at for Tanimoto similarity calculations. A graphical representation of the degree of similarity among the compounds is presented as a heatmap, as shown in Figure 2. The diagonal yellow line from the top left corner to the bottom right indicates the comparison of each molecule with itself, resulting in the highest similarity score of 1.0, as shown by the colour scale on the right. The colour scale ranges from purple, indicating minimal similarity (0.0), to yellow, signifying significant resemblance (1.0). The heatmap is predominantly purple, indicating that most molecules show minimal similarity to each other. Some green to yellow squares are scattered, showing areas where certain molecules share more similarities. The clustering algorithm further analysed the substances by grouping them according to their similarity in a multidimensional space. Furthermore, Figure 3 shows the k means clustering plot, in which centroids are marked with a red X sign. Clustering showed three distinct clusters, in which two clusters formed a closed cluster, which suggested a compact cluster showed decreased variability among the data points within that group. The homogeneity of similar molecules can be highly advantageous in applications that require the prediction of their behaviour or attributes, as the close grouping indicates reliable and constant characteristics. One cluster on the axis showed a scattered cluster, which indicates a higher degree of variability among the data points within that cluster. Therefore, the molecule at the centroids serves as the true representative of each cluster. Here, the three centroids (5297, 432449, and 85137543), each representing a molecule from a cluster, were selected for the MD simulation.

**Figure 4:**
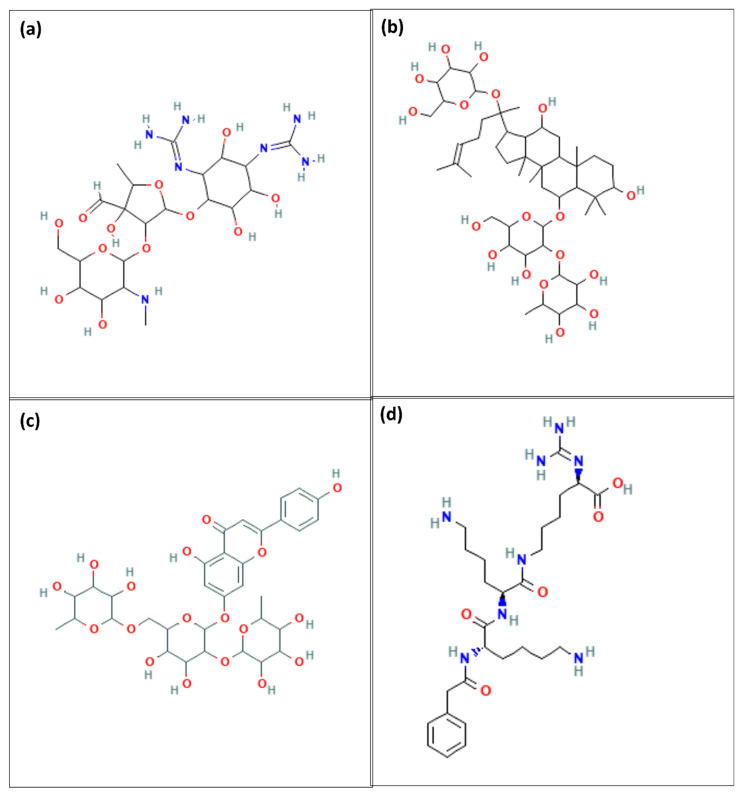
Two-dimensional structure representations of the three selected compounds, (**a**) 5297, (**b**) 432449, (**c**) 85137543, and (**d**) control.

In this study, binding energies of 205 compounds were evaluated, normalising the scores to ensure accurate comparison, as detailed in Table S1. From this analysis, 28 compounds were selected that demonstrated superior binding affinities compared to the control compound, with binding energies ranging from −7 kcal/mol to −9 kcal/mol and normalised scores between −6.8 kcal/mol and −8.6 kcal/mol. To further explore these compounds, three structurally diverse representatives were selected by employing a clustering approach based on the Tanimoto similarity index. This method allowed us to identify compounds that served as centroids of their respective clusters, thus ensuring a broad representation of chemical diversity. The three selected compounds—5297, 432449, and 85137543—underwent molecular dynamics simulations, providing insights into their potential as therapeutic agents. The 2D structures of the selected compounds are shown in Figure 4.

### 2.5. MD Simulation

#### 2.5.1. RMSD

**Figure 5:**
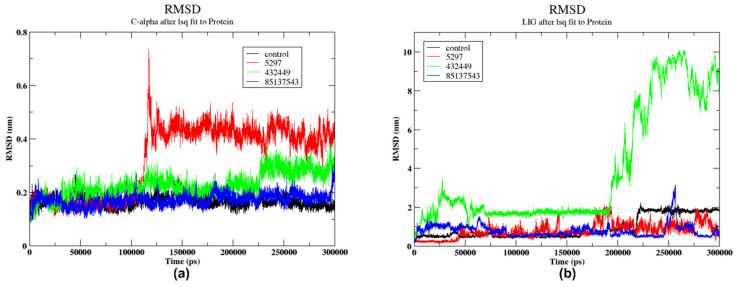
RMSD trajectories for proteins and ligands under different conditions (**a**) represent the protein RMSD and (**b**) represent the RMSD of the ligand.

**Figure 6:**
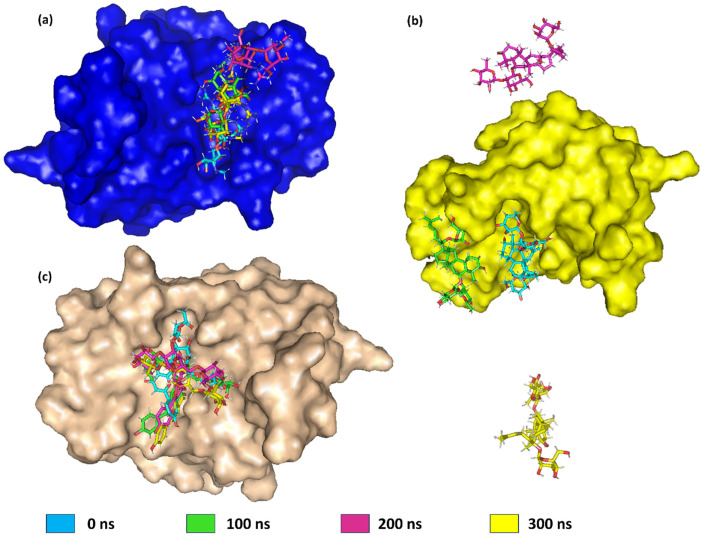
Molecular visualisation of a protein with bound ligands in different configurations 0 ns (cyan), 100 ns (green), 200 ns (magenta), and 300 ns (yellow). The protein–ligand complexes for (**a**) 5297, (**b**) 432449, and (**c**) 85137543.

Figure 5a shows the root mean square deviation (RMSD) of the protein. The RMSD values fluctuate significantly, ranging from around 0.05 to 0.25 nm, indicating substantial conformational changes or structural deviations from the reference structure during the simulated or analysed time period. Moreover, the protein molecule associated with the control compound showed significant initial fluctuation in the RMSD, which peaks at around 50 ns. This might indicate the protein is settling into a stable conformation after starting from an unstable state. The protein 5297 ligand complex exhibits minor fluctuations initially, but at 100 ns, it experiences a significant increase of 0.7 nm, followed by a stable confirmation. The protein molecule 432449 shows moderate fluctuations, generally between 0.2 nm and 0.4 nm, indicating noticeable but less extreme structural changes. Moreover, the protein molecule 85137543 shows maintaining the lowest RMSD values, which rarely exceed 0.2 nm, indicating a stabilised structure. Overall, this suggested that 85137543 showed more stable confirmation compared to other compounds. Moreover, when aligning the protein molecule, the RMSD for the ligand was calculated, and the outcome is shown in Figure 5b. The control condition exhibited distinct deviations reaching 0.55 nm at 48 ns and 60 ns, followed by a relatively stable conformation up to 150 ns. Subsequent to this period, moderate fluctuations ranging from 0.55 nm to 1.25 nm were observed. A noticeable increase occurred at 225 ns, where the RMSD reached 2 nm. Thereafter, the system returned to a stable conformation for the final 10 ns of the simulation. RMSD of compound 5297 fluctuates between 0.1 nm and 2 nm. There are several noticeable peaks throughout the simulation around 100 ns, 125 ns, and 200 ns, as shown in Figure 5b and Figure 6, that suggest instants where the ligand undergoes substantial deviations from its initial conformation.

A previous study conducted a 10 ns simulation, which indicated that control exhibited high rigidity throughout the simulation [18]. This rigidity was attributed to an intramolecular ionic interaction between its charged termini and was supported by RMSD values that consistently remained below 2.2 Å (0.22 nm) over the 10 ns simulation, with minimal shifts in torsion energies, indicating a stable and rigid binding conformation. The torsion energy analysis in the previous study showed a sharper distribution of energies in the bound state, highlighting significant rigidification upon complex formation. In contrast, this study extended the simulation to 300 ns, revealing a more dynamic picture of compound 4 over the longer period. Initially, this study’s findings align with the previous research, showing that control-maintained stability with an RMSD below 0.55 nm up to 60 ns, corroborating the earlier report of its initial rigidity.

Furthermore, the ligand variations deviate significantly from their original values at key time intervals (0 ns, 100 ns, 200 ns, and 300 ns), as seen in Figure 6. Figure 6a showed the initial position of 5297 when bound to the protein (cyan). At 100 ns, 5297 was observed to be present in the pocket (green), while at 200 ns (magenta), it moved from the pocket to the surface. Later, at 300 ns, it returned to the pocket (yellow). The RMSD value of the ligand 432449 initially showed low fluctuations around 2 to 3 nm. However, after 200 ns, it moved out of the complex, as shown in Figure 6b. This increasing trend in RMSD implies that the ligand undergoes significant conformational changes or positional shifts over time, moving farther away from its preliminary bound state in the protein binding site or cavity. The large RMSD values towards the end indicate a high degree of deviation from the reference ligand–protein structure. Therefore, this ligand was not used in further analysis. The RMSD value of the ligand 85137543 showed stable confirmation at the primary 50 ns. Later, the RMSD values fluctuate considerably, with multiple peaks observed, such as at 200 ns and at 300 ns, as shown in Figure 5b. This indicates the ligand repeatedly deviates from and returns closer to the reference binding pose or conformation within the protein. Moreover, in Figure 6c, variation in the ligand position can be observed at 100 ns, 200 ns, and 300 ns. This shows that at 100 ns, 200 ns, and 300 ns, the compound had the same orientation in binding to the protein, thus suggesting strong binding to the protein.

#### 2.5.2. RMSF and SASA

**Figure 7:**
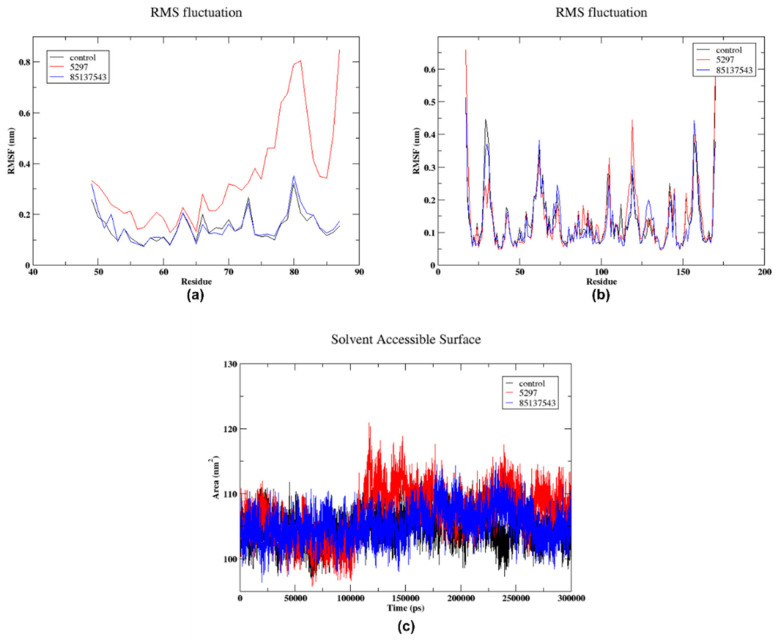
(**a**) RMSF of the chain A and (**b**) RMSF of the chain B. (**c**) The SASA plot showed a change in exposed surface area with simulation time in picoseconds (on-axis).

Figure 7a shows the RMSF of chain A. The ligand 5297 exhibited minimal variations in the initial residues (about residues 40 to 70), resembling the control. This indicates that the presence of this ligand does not have a substantial impact on the flexibility of these specific regions of the protein. Nevertheless, there is a prominent spike observed between residues 80 to 90, where the RMSF value reaches roughly 0.3 nm to 1 nm, suggesting a substantial level of flexibility or mobility in these specific residues, maybe attributed to the impact of the ligand. Ligand 85137543 shows higher RMS variations, particularly around residues near 60 and 80–85. While the control simulation generally exhibits lower RMS fluctuations across most residues compared to the other two conditions, Figure 7b shows the RMSF of chain B. The control showed fluctuations at values of 0.3 nm at residues 62–86, 157–158. Similarly, ligands 5297 and 85137543 exhibited fluctuations exceeding 0.3 nm at residues 29–34, 62–66, and 158–161. The ligand 8,513,543 exhibits larger RMSF values compared to the control entity 5297, particularly in specific regions of the residue range. Nevertheless, there are certain areas where the lines overlap or connect, suggesting that the RMSF values are comparable for both ligands in those places. Figure 7c shows the solvent-accessible surface area (SASA) of the protein. The control remains relatively consistent throughout the simulation, indicating stable exposure of the protein surface to the solvent without the influence of ligands. Ligand 5297 shows greater fluctuations in SASA compared to the control. The SASA frequently spikes above the control level and dips below it, suggesting that the binding or presence of this ligand induces significant conformational changes in the protein. These changes either expose more of the protein to the solvent or cause the protein to fold in a way that shields more surface area. Ligand 85137543 The SASA for this ligand generally remains lower than the control, suggesting that this ligand causes the protein to adopt a conformation that consistently shields more of its surface from the solvent compared to the control. However, it shows fewer and lower amplitude fluctuations than the 5297, indicating less variability in conformation compared to the 5297.

Overall, the SASA results indicate that both ligands affect the protein’s conformation differently; ligand 5297 appears to cause more dynamic and variable conformational changes, as evidenced by the higher and more frequent fluctuations in SASA. Ligand 85137543 generally leads to a more consistent reduction in solvent-exposed surface area, suggesting a stabilising effect on the protein’s conformation with less exposure to the solvent.

#### 2.5.3. Hydrogen Bond

**Figure 8:**
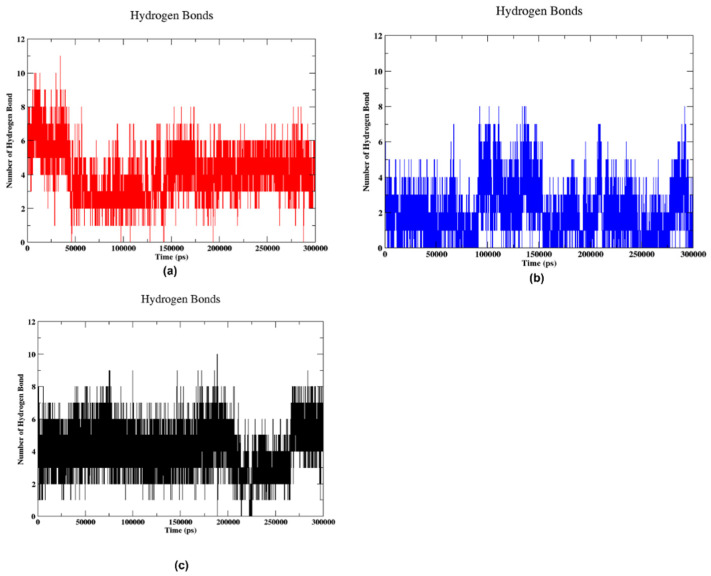
Hydrogen formation during 300 ns trajectory for (**a**) 5297, (**b**) 85137543, and (**c**) control.

Hydrogen bonding is a crucial interaction for stabilising the protein–ligand complex. Figure 8 shows the number of hydrogen bonds formed in the complexes along the 300 ns trajectory. When ligand 5297 was bound to the protein, it formed 8 to 11 bonds in the initial 48 ns, as shown in Figure 8a. Later, the number of bonds decreased by 4 at 50 ns, then gradually decreased from 55 ns to 148 ns, reaching 5–7 bonds. After 150 ns, it established the 7–8 bond, which lasted until 300 ns. The variability implies a changing relationship in which the frequency of hydrogen bond development fluctuates, potentially indicating less stable binding or substantial conformational changes within the molecular complex. In Figure 8b, the hydrogen bond formation between the ligand 85137543 and the protein exhibited dynamic behaviour throughout the simulation. Initially, it formed five stable hydrogen bonds within the first 50 ns. Subsequently, the number of bonds increased to 7 around 60 ns, followed by a decrease to 3–4 bonds. Between 80 ns and 150 ns, the ligand formed a maximum of eight hydrogen bonds with the protein. After that, the bond count was reduced again to 4–5 bonds in the 150–200 ns timeframe. At around 220 ns, the number of bonds rose again, forming 6–7 bonds to 250 ns. Then, it decreased to 3–4 bonds before rising once more from five to eight bonds during the final 20 ns of the simulation. This suggests it exhibits variations; however, there are periods during which the quantity of hydrogen bonds remains consistently low, indicating phases of reduced interaction. The peaks represent intervals of higher interaction, characterised by a substantial rise in the number of hydrogen bonds followed by a subsequent decline. Figure 8c showed the formation of hydrogen bonds when control bound to the protein initially; up to 65 ns, a stable formation of eight hydrogen bonds was observed. Subsequently, around 70 ns, the number of bonds increased by one, reaching 9. From 80 ns to 170 ns, the control maintained a consistent formation of 8–9 hydrogen bonds with the protein. At 180 ns, there was a temporary rise, with one additional hydrogen bond formed. However, after this point, a reduction occurred, resulting in the formation of 6 hydrogen bonds from 220 ns to 260 ns. Finally, from 260 ns until the end of the simulation at 300 ns, a stable configuration of eight hydrogen bonds was observed. The distribution of hydrogen bonds in this graph is more consistent and denser compared to the others. The number of hydrogen bonds appears to remain relatively high and stable, fluctuating between about 4 and 10, indicating a more stable interaction or a smaller degree of conformational change in the molecular complex.

**Figure 9:**
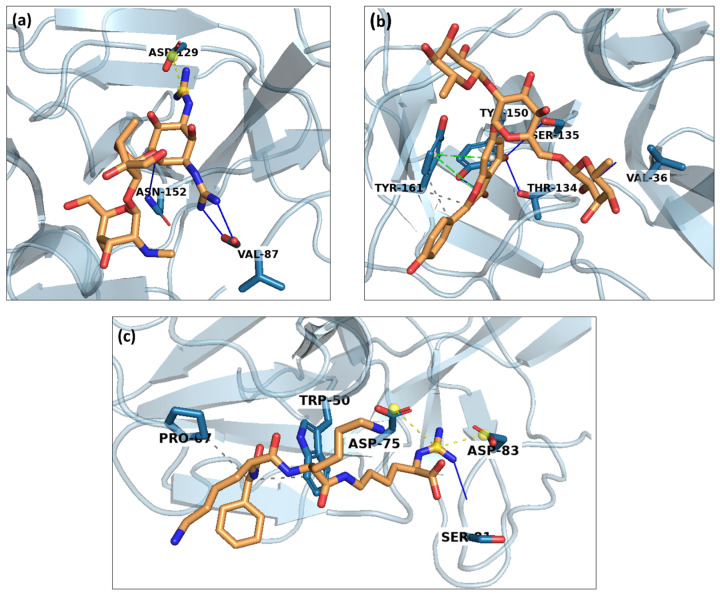
Interaction analysis for the protein–ligand complexes of (**a**) 5297, (**b**) 85137543, and (**c**) control.

In the molecular dynamics analysis of the ZIKV NS2B/NS3 protease–ligand complexes, distinct interaction profiles were observed across different compounds. For complex 5297 (Figure 9a), key residues such as ASN129, ASN152, and VAL87 play a crucial role in stabilising the ligand through hydrogen bonds and hydrophobic interactions, respectively. Meanwhile, complex 85137543 (Figure 9b) demonstrates significant interactions with residues TYR150, SER135, and TYR161, indicating a combination of hydrogen bonding and pi-stacking that potentially enhances ligand binding affinity. It also shows interaction with THR134 and VAL36. In contrast, the control complex (Figure 9c) exhibits unique interactions primarily involving residues TRP50 and ASP75, suggesting a mix of pi-stacking and ionic interactions. The control’s ligand conformation is likely maintained by a network of hydrogen bonds involving SER81 and ASP83. It also shows interaction with PRO67. These interaction differences underscore the diverse binding mechanisms and conformational dynamics across the complexes, highlighting the structural adaptations that contribute to their binding affinity and specificity. The distinct interactions observed for each compound provide insights into their stability and potential efficacy as inhibitors, which could inform future drug design and optimisation efforts.

The alignment of the protein–ligand complexes for compounds 5297 and 85137543 over the crystal structure of 7ZMI is depicted in Supplementary Figure S1. The analysis revealed an RMSD (root mean square deviation) of 0.8 Å for compound 5297 and 0.7 Å for compound 85137543, indicating a high degree of structural similarity and alignment accuracy with the 7ZMI reference structure. These low RMSD values suggest that both compounds closely mimic the binding orientation of the known inhibitor in the NS3 protease active site, highlighting their potential as effective inhibitors.

#### 2.5.4. PCA and FEL

**Figure 10:**
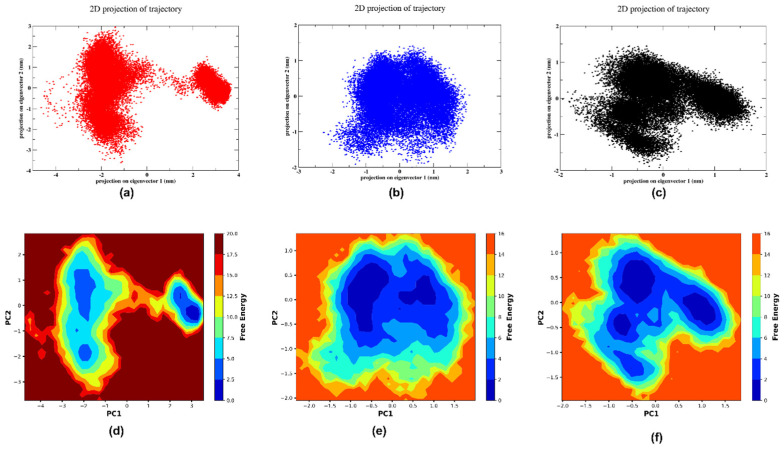
The scatter plot of the PCA for (**a**) 5297, (**b**) 85137543, and (**c**) control and the free-energy landscape of (**d**) 5297, (**e**) 85137543, and (**f**) control.

The principal component analysis (PCA) shows the trajectory that was created by the MD simulation as a scatter plot. As shown in Figure 10a, molecule 5297 formed two separate clusters during the 300 ns simulation, which showed the transition from the initial state to the final state. Molecule 85137543 formed a single concentrated cluster, as shown in Figure 10b. This indicated that the range of possible conformations examined is more evenly distributed and focused on a particular state. Similarly, the control formed a single cluster with a lesser scattered form that showed a low degree of transition, as shown in Figure 10c. Moreover, Figure 10d shows the plots of 5297 that show there are three energy basins with distinct energy barriers, indicating stable configurations of the system that are separated by barriers in space. In addition, Figure 10e shows the plot of 85137543, which formed two energy basins with a single energy barrier. The presence of these two energy basins suggests that the system potentially has two distinct low-energy conformational states. The central, deeper basin likely corresponds to the global minimum or most stable state, while the smaller basin may represent a local minimum or a less favourable but still relatively stable state. Furthermore, Figure 10f shows the plot of control, which formed three distinct energy barriers with significant energy barriers. The central basin has greater dimensions and depth, shown by a darker shade of blue, and is encompassed by elevated energy barriers represented by the colours orange and red. This suggests a condition of greater stability, characterised by reduced fluctuation and a decreased likelihood of transitioning to higher energy states. The system appears to be highly optimised for maintaining this specific conformation, necessitating a considerable amount of energy to deviate from this state. Overall, molecule 5297 has notable structural flexibility, while molecule 85137543 exhibits a more stable behaviour, primarily maintaining a single conformational state, which can be beneficial for drug design.

#### 2.5.5. MM/GBSA

**Figure 11:**
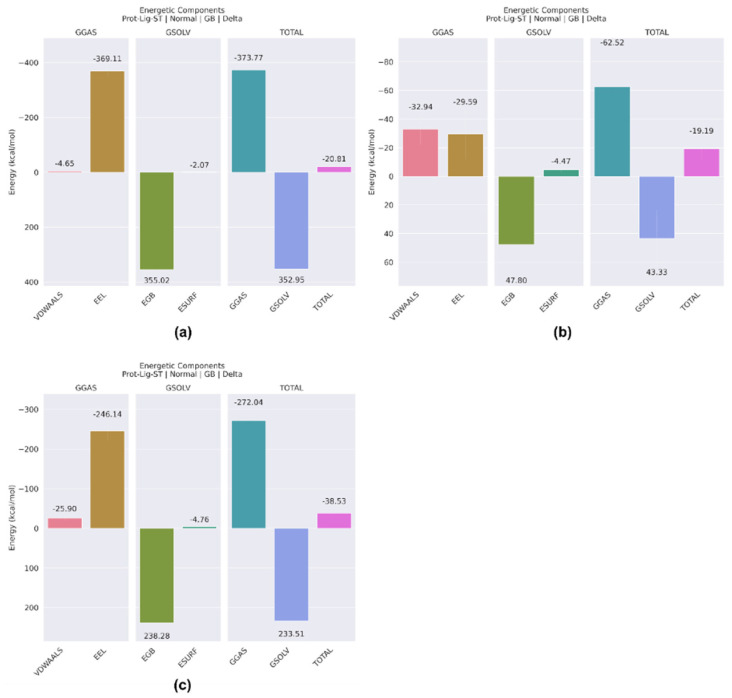
The representation of the complex of the molecule based on binding free energy using the MMGBSA approach for (**a**) 5297, (**b**) 85137543, and (**c**) control.

Molecular mechanics with generalised Born and surface area solvation (MM/GBSA) is a widely used approach for determining the free energy associated with the binding of ligands to proteins [19]. Here, Figure 11a shows that the overall Gibbs free binding energy of the 5297 complex is −20.81 kcal/mol, indicating a favourable binding interaction between the protein and the ligand. Figure 11b shows the total binding energy of the 85137543 complex, which is −19.19 kcal/mol, which suggests a positive binding affinity between the protein and the ligand. The presence of a negative total value indicates that the energy changes in the system favour the formation of a stable protein–ligand interaction. Moreover, a total binding energy of −38.53 kcal/mol was observed in the control complex, suggesting strong affinity for the protein molecule. Overall, it was observed that complex 5297 showed comparable binding energy with protein molecules and emerged as the best fit that could bind with the target protein.

### 2.6. Compound Properties

ADMET properties calculations of the compounds were performed using SwissADME [20] and ProTox 3.0 [21] as listed in Table 2. Compounds 5297 and 85137543 exhibit a high soluble nature and thus facilitate rapid distribution in extracellular fluids. Both belonged to the acceptable toxicity class of 3 and 5 based on the Globally Harmonised System of Classification and Labelling of Chemicals (GHS) and the Hazard Communication Standard (HCS). The 85137543 exhibits higher lipophilicity (iLOGP of 0.14) compared to 5297, likely leading to a larger volume of distribution and higher protein binding. Both showed no PAINS (Pan-Assay INterference compounds) alert, suggesting that the compounds are more likely to have specific interactions with their intended targets, leading to more reliable experimental results and a higher likelihood of success in therapeutic applications. These findings indicate drug-like properties of 5297 and 85137543, in particular regarding their potential for oral bioavailability.

Compound 5297 (Streptomycin) is an aminoglycoside antibiotic primarily used to treat bacterial infections, especially those caused by Gram-negative bacteria [22]. Its mechanism of action involves binding to the 30S subunit of bacterial ribosomes, leading to misreading of mRNA and inhibiting protein synthesis, ultimately exerting a bactericidal effect. Compound 5297 (Streptomycin) is particularly effective against *Mycobacterium tuberculosis* and is used in treating tuberculosis [23]. Compound 5297 (Streptomycin) does not directly interact with viral proteins like NS3 protease, as its primary target is bacterial ribosomes. On the other hand, 85137543 (5-Hydroxy-2-(4-hydroxyphenyl)-4-oxo-4H-chromen-7-yl 6-deoxy-alpha-L-mannopyranosyl-(1->2)-[6-deoxy-alpha-L-mannopyranosyl-(1->6)]-beta-D-glucopyranoside) is a flavonoid glycoside known for its antioxidant, anti-inflammatory, and potential antimicrobial properties. Flavonoids like this compound (85137543) are renowned for scavenging free radicals, reducing oxidative stress, and inhibiting inflammatory pathways, which may provide a degree of antimicrobial and anti-cancer activity. Previous studies have shown the Flavonoids to act as allosteric inhibitors of Flavivirus NS2B-NS3 protease [24]. Flavonoids are known to have antiviral activities targeting Zika virus (ZIKV), Dengue virus (DENV), Hepatitis C virus (HCV), Influenza virus (SARS-CoV-2), Herpes Simplex virus (HSV), and Human Immunodeficiency virus (HIV) [25,26,27]. They are found to interact with NS5 polymerase, NS4B protein, HIV-1 reverse transcriptase and protease, main protease (Mpro), and RNA-dependent RNA polymerase (RdRp) [27]. Overall, both compounds have distinct biological activities and therapeutic applications, with Streptomycin primarily targeting bacterial infections and the flavonoid glycoside exhibiting broader biochemical effects, including potential interactions with cellular enzymes and signalling proteins.

In addition to its well-known antibacterial activity, this study has identified Streptomycin as a drug that targets and inhibits the NS3 protease of the Zika virus, which is crucial for viral replication. This interaction highlights Streptomycin’s potential as an antiviral agent against Zika, expanding its therapeutic applications beyond bacterial infections.

### 2.7. Discussion

In this study, we focused on identifying potential inhibitors of the Zika virus (ZIKV) NS2B-NS3 protease using an in silico approach. The Zika virus, a member of the Flavivirus genus, has emerged as a significant global health concern due to its rapid spread and severe health implications, including congenital abnormalities in newborns and neurological disorders in adults. The lack of specific antiviral treatments and vaccines underscores the urgent need for novel therapeutic approaches. This study utilised a machine learning-based QSAR model to screen 2864 natural compounds, ultimately identifying three promising candidates: 5297 (Streptomycin), 432449, and 85137543 (5-Hydroxy-2-(4-hydroxyphenyl)-4-oxo-4H-chromen-7-yl glycoside). Among these, 5297 exhibited the most stable interaction with the NS3 protease, as indicated by the consistent formation of hydrogen bonds and minimal root mean square deviation (RMSD) during a 300 ns molecular dynamics simulation. The binding free energy calculation using the MM/GBSA method further confirmed Streptomycin’s superior binding affinity, with a ΔG of −20.81 kcal/mol.

Previous studies provide valuable insights that complement our findings. For instance, a recent study conducted a structure-based virtual screening and molecular dynamics simulation to identify potential inhibitors for the ZIKV NS3 helicase protein, another crucial component of the viral replication machinery [28]. This study identified ZINC01033978 as a top inhibitor based on GlideGscore, emphasising the importance of targeting the NS3-Hel protein due to its role in viral replication. Similar to our findings, the study highlights the significance of hydrophobic interactions and hydrogen bonding in achieving strong binding affinity with the target protein. The identified inhibitors demonstrated compliance with Lipinski’s rule of five, indicating good drug-likeness and pharmacokinetic properties. Another study employed a structure-based approach to develop a Pharmacophore Anchor (PA) model for the ZIKV NS3 protease active site. This model identified 12 anchor points across the protease’s active site, with five core anchors being conserved across flaviviral proteases. This study successfully identified FDA-approved drugs Asunaprevir and Simeprevir as potent inhibitors of the NS3 protease, highlighting the potential for repurposing existing drugs for anti-ZIKV treatments [10]. The identified drugs exhibited IC50 values of 6.0 µM and 2.6 µM, respectively, demonstrating significant anti-ZIKV activity. This aligns with our findings, where Streptomycin also showed strong binding interactions within the NS3 protease active site. In a separate study, 26 compounds were screened against the ZIKV NS2B-NS3 protease, with eight showing significant inhibitory activity. Among these, one compound displayed whole-cell anti-ZIKV activity, further validating the potential of targeting the NS3 protease for antiviral drug development [29]. This study emphasises the relevance of using structure-based approaches to identify lead compounds for ZIKV treatment. Furthermore, the comparison of these compounds with existing NS3 protease inhibitors, such as those described in the structure 7ZMI, provides a benchmark for understanding their potential effectiveness. The inhibitors in 7ZMI, such as MI-2113, demonstrate specific binding interactions with the NS3 protease, including hydrogen bonding and hydrophobic interactions, which could serve as a model for optimising the binding affinity of 5297.

While this study’s in silico findings are promising, experimental validation is necessary to confirm the efficacy of Streptomycin and the other identified compounds. In vitro and in vivo assays are essential to evaluate the antiviral activity and pharmacokinetic properties of these inhibitors.

## 3. Material and Methods

### 3.1. Protein Structure and Compound Library Preparation

The protein 3D crystal structure of NS2B_NS3 protease was obtained from the protein data bank (PDB) using the PDB ID: 7ZMI [18]. Additionally, the ligand’s co-crystal structure, which is a recognised inhibitor compound, MI-2113, has been linked to the protein [30] and was used as a control in further studies. Moreover, chain A and chain B were both used. Later, the SelleckChem natural product library (https://www.selleckchem.com/screening/natural-product-library.html (accessed on 4 April 2024)) was used to screen the natural compounds, resulting in a total of 2864 natural compounds. In addition, these compounds were analysed using a QSAR-based machine learning model.

### 3.2. Machine Learning-Based QSAR Model

Six regression models were employed for modelling the activity of compounds, including linear regression, random forest regressors, Bayesian ridge regression, decision tree regressors, support vector regression, and gradient boosting regression. Subsequently, searching was performed on the ChEMBL database [31] at https://www.ebi.ac.uk/chembl/ (accessed on 12 August 2024) to collect the compounds needed for developing the prediction models. The keyword “Zika” was searched in the ChEMBL database, which led to the identification of a single target ID that represented the whole organism. Compounds with an EC50 activity score were specifically targeted and selected for further model building. Later, the RDkit package version 2024.03.5 was used to calculate the SMILES notation of the compounds. Subsequently, the compounds were selected based on their EC50 values and then normalised using logarithm base 10. Afterward, the length of the SMILES sequence was also ascertained. Following that, 30% of the selected compounds have been designated for assessing the model’s predicted performance, while the remaining 70% were utilised for training the model. The coefficient of determination (R^2^) was calculated to ensure the quality of the training models. The model with the highest R^2^ value was selected as the most robust and accurate predictive model and was used in the following screening. Moreover, this trained model was used to perform QSAR analysis on the obtained natural compound and control ligand, and based on the control EC50 value, compounds were selected.

### 3.3. Molecular Docking

The protein–ligand molecular docking was performed using the Autodock tool version 4.2.6 [32]. Subsequently, the grid box was formed, covering the binding site residues with the following dimensions for the NS3 protein: 22 Ǻ × 24 Ǻ × 28 Ǻ with the centre at −7.71 × 0.21 × −14.78 along the x, y, and z axes, respectively. The protein molecule was added with hydrogen atoms and thereafter constructed by allocating Kollamn charges and partial charges to each atom. Water molecules and heteroatoms are subsequently removed prior to docking. Furthermore, during docking-based virtual screening, the following parameters were considered: 20 binding modes, 100 exhaustiveness, and a maximum energy difference of 4 kcal/mol. Later, the selected ligands have been normalised following the equation:

average=Average of (Binding Energy/Top Binding Energy)

Normalized Score=Top Binding Energy ∗ average

### 3.4. Tanimoto Similarity and Clustering

In the present study, the docked compounds were compared with the control using Tanimoto similarity. The Tanimoto similarity between all pairs of molecules is calculated using Morgan fingerprints from RDKit version 2024.03.5. A similarity matrix is created from the Tanimoto similarities, and a heatmap visualisation is generated using Seaborn. Followed by clustering analysis using the K-means clustering algorithm [33]. The centroids (compounds) were found in the acquired cluster, and further were selected for molecular dynamic simulation along with a control.

### 3.5. Molecular Dynamic (MD) Simulation

The most populated cluster of the compounds was selected for the MD simulation, and the 300 ns simulation was performed using Gromacs version 2022.1 [34]. Subsequently, the molecular topologies formed by the CHARMM36 version 50 [35] force field parameters. The top compounds and the control inhibitor’s force field characteristics were developed using the CGneFF server version 4.6 [36]. Afterwards, the electrostatic force transversely over a specific distance was calculated using the Ewald particle mesh method [37]. The system was hydrated using the TIP3P model and put into a cubic solvation box that was 1.0 nm from the wall [38]. Afterward, the ions Na^+^ and Cl^−^ were employed to neutralise the system. To eliminate steric conflicts, the system used the steepest descent (SD) method for 50,000 iterations. Later, the LINCS [39] algorithm has been used to enforce constraints on the bonds and achieve system stability. Moreover, the system was brought up to 310 K over a simulation period of 100 ps using a timestep of 2 fs in the NVT ensemble. In addition, the system was initially equilibrated by applying constant pressure (NPT ensemble) for a duration of 1 nanosecond (ns) at 310 Kelvin (K) and 1 atmosphere of pressure. Subsequently, the primary molecular dynamics simulation was carried out for a production run lasting 300 nanoseconds. During the production run, the Parrinello–Rahman pressure coupling method [40] was employed to maintain a constant pressure, while the velocity-rescaling [41] thermostat was utilised to couple and regulate the temperature of the system. Later, a post-simulation analysis was performed using the built-in tools in GROMACS to evaluate the following properties: root mean square deviation (RMSD), which measures the deviation of the simulated structure from a reference structure; root mean square fluctuation (RMSF), which quantifies the fluctuations of individual atoms or residues from their average positions; and hydrogen bonding interactions within the system.

### 3.6. PCA

PCA can be employed to analyse and interpret large datasets containing information about drugs, their chemical structures, and their biological activities. PCA allows for the reduction in high-dimensional data into a smaller number of principal components, which capture the most significant variations within the dataset [42]. The trajectory was preprocessed for principal component analysis by removing the periodic boundary condition. The Gmx_covar tool, which is part of GROMACS, was used to calculate the covariance matrix. The covariance matrix represents the relationship between the atomic fluctuations of the protein–ligand combination. The GROMACS utility ‘gmx anaeig’ was utilised to compute the eigenvalues and eigenvectors of the covariance matrix. Subsequently, the ‘gmx anaproj’ tool from the GROMACS suite was employed to calculate the principal component (PC) coordinates for each frame of the trajectory. This step facilitated the visualisation of the trajectory projected onto the principal components, enabling the exploration of the conformational space sampled during the simulation.

### 3.7. Free-Energy Landscape

The analysis of the free-energy landscape (FEL) elucidates steady-state and kinetic aspects of biological systems. Investigation of FEL minima and barriers yields automatic insights into pivotal phenomena, including biomolecular recognition, aggregation, and folding processes. Identification of energetically favourable conformational basins and transition state ensembles facilitates understanding of the thermodynamic stabilities and kinetic pathways governing this critical biomolecular process [43]. To determine the FEL, the energy distribution was computed as shown by the following equation:

$$∆G\left(X\right)=-kBTlnP(X)$$

The Gibbs free energy is denoted by the symbol G. The Boltzmann constant, represented by kB, is a fundamental physical constant relating energy to temperature. T symbolises the absolute temperature. X signifies the reaction coordinate, which is a collective variable or descriptor that captures the progress of a reaction or transition process. P(X) represents the probability distribution function of the system along the reaction coordinate X, describing the likelihood of observing the system in a particular state or configuration along the reaction path.

### 3.8. MM/GBSA

The MM/GBSA method is frequently used to identify novel matches in virtual screening campaigns. This method is expected to enable more reliable assessments of ligand-binding affinities compared to the basic score systems used in molecular docking algorithms [44]. Here, the Gibbs free binding energy of the protein–ligand complex was determined using the GROMACS add-on tool gmx MM/PBSA [45]. Equations (1)–(6) are used to calculate the MM/GBSA.

(1) $$∆G=G_{complex\;}-[G_{receptor}+G_{ligand}]$$

(2) $${\Delta G}_{binding}=\Delta H-T\Delta S$$

(3) $$\Delta H=\Delta G_{GAS}+\Delta G_{SOLV}$$

(4) $$\Delta G_{GAS}=\Delta E_{EL}+\Delta E_{VDWAALS}$$

(5) $$\Delta G_{SOLV}=\Delta E_{GB}+\Delta E_{SURF}$$

(6) $$\Delta E_{SURF}=\gamma .SASA$$

Equation (1) defines Δ*G* as a change in Gibbs free energy during the protein–ligand synthesis. The total free energies of the protein–ligand complex, free enzyme, and ligand in the solvent are represented by $G_{complex}$, $G_{receptor}$, and $G_{ligand}$, respectively. ${\Delta G}_{binding}$ denotes the change in Gibbs free energy for the binding interaction between protein and ligand molecules, while ΔH represents the change in enthalpy that includes the sum of the gas-phase energy (ΔG_GAS_) and the total solvation free energy (ΔG_SOLV_). The binding free energy, shown as TΔS, is the cumulative sum of the change in entropy. ΔE_EL_ represents a change in electrostatic energy, while $\Delta E_{VDWAALS}$ signifies the change in van der Waals energy. These two components combine to yield $\Delta G_{GAS}$. Furthermore, $\Delta E_{SURF}$ represents a change in solvation-free energy caused by the non-polar interaction, while ΔE_GB_ represents the change in polar solvation energy by polar group interaction. The calculation of $\Delta E_{SURF}$ involved the change in the solvent-accessible surface area (SASA) by the solvent surface tension parameter (γ).

## 4. Conclusions

The objective of the current study was to determine the potential target that inhibits the action of the Zika virus infection. Therefore, the in silico pipeline was made to identify the potential new NS2B_NS3 protease-specific inhibitors. Hera, a small molecule from the natural product library, was screened, resulting in three promising candidates from the top hits, identified as 5297, 432449, and 85137543. During the evaluation, it was noted that compound 432449 dislocated from the binding site and was subsequently excluded from further analysis. The remaining two compounds, however, demonstrated consistent behaviour. Compound 5297 was the most promising candidate for further investigation as a potential inhibitor of the Zika virus NS3 serine protease, based on its favourable binding characteristics and structural stability observed through the integrative computational approach.

## Figures and Tables

**Table 1:** Validation metrics for the Trained Model Coefficient of Determination (R^2^) values.

Model	R^2^
Bayesian Ridge	0.61
Linear Regression	0.61
Random Forest Regressor	0.79
Decision Tree Regressor	0.54
Support Vector Regression	−0.57
Gradient Boosting Regression	0.77

**Table 2:** ADMET properties of the compounds 5297 and 85137543.

Molecule	5297	85137543
MW	581.57	724.66
Rotatable bonds	9	8
H-bond acceptors	15	18
H-bond donors	12	10
MR	130.43	168.55
TPSA	336.43	287.89
iLOGP	−0.06	0.14
Solubility	Highly soluble	Soluble
PAINS alerts	0	0
Toxicity	3	5

## Data Availability

Data are contained within the article.

## Supplementary Materials

The following supporting information can be downloaded at: https://www.mdpi.com/article/10.3390/ph17081067/s1, Table S1: Top binding score and normalized binding scores from docking for the selected ligands. Figure S1. Structure of the protein-ligand complex of 5297 and 85137543 aligned over the crystal structure of 7ZMI.
