# Supplementary material for: Employing Machine Learning-Based QSAR for Targeting Zika Virus NS3 Protease: Molecular Insights and Inhibitor Discovery
Source: Pharmaceuticals (Basel). 2024 Aug 15;17(8):1067. doi: 10.3390/ph17081067 (PMC11359100; doi:10.3390/ph17081067)
Supplement: Supplementary file 1 [file pharmaceuticals-17-01067-s001.zip › pharmaceuticals-3132508-supplementary.pdf]

Table S1. Top binding score and normalized binding scores from docking for the selected ligands.

Top	Compound	Normalized Binding Score
-9.122	6442411	-8.67967
-9.145	115063	-8.49645
-8.4	85137543	-8.16666
-8.825	6124213	-8.08589
-8.276	12315188	-7.98267
-8.536	14413436	-7.98122
-8	73826569	-7.78889
-8.174	137796485	-7.749
-8.501	108062	-7.71511
-8.118	14353383	-7.52011
-8.039	13880097	-7.45022
-8.171	14284443	-7.44323
-8.396	161823	-7.32022
-7.661	15115604	-7.20678
-7.783	320761	-7.16245
-7.809	2142	-7.14189
-7.607	432448	-7.13322
-7.924	2996	-7.125
-7.801	14284432	-7.11766
-7.871	432449	-7.108
-7.2	85085043	-7.1
-7.396	14525328	-7.04445
-7.537	124222337	-7.03
-7.508	5297	-7.02311
-7.404	5360237	-7.01578
-7.572	432450	-6.99033
-7.826	320760	-6.93922
-7.185	4689	-6.86378
-7.361	control_166175727	-6.84922
-7.25	4483043	-6.84578
-7.093	226371	-6.84145
-7.172	100018	-6.82133
-7.487	4432690	-6.80878
-7.36	5513	-6.808
-7.224	4471696	-6.76022
-7.016	13375991	-6.75522
-7.781	5891232	-6.75022
-6.934	13370031	-6.72456

-8.073	3069	-6.68934
-7.248	15574810	-6.68022
-7.043	432524	-6.67333
-7.179	10462625	-6.62322
-6.91	3755	-6.60389
-7.236	5772	-6.55511
-6.998	354578	-6.54267
-7.438	139600636	-6.53278
-6.918	4483635	-6.50733
-6.843	4071695	-6.50467
-6.676	50587	-6.43
-6.853	317	-6.413
-6.647	156610575	-6.38889
-6.785	5087640	-6.38378
-6.6	75061243	-6.37778
-6.771	4287569	-6.34844
-6.622	6370943	-6.32855
-6.967	4277538	-6.26367
-6.562	4454	-6.25478
-6.584	3707011	-6.209
-6.355	117709930	-6.18222
-6.858	439519	-6.15844
-7.325	15631736	-6.12622
-6.7	74977499	-6.12223
-6.445	2973	-6.10589
-6.712	3258	-6.01122
-6.668	5184	-5.92922
-5.997	4607242	-5.75756
-6	815	-5.75555
-5.9	75585866	-5.68889
-6.578	5458486	-5.40956
-5.6	74029749	-5.32222
-5.6	853	-5.27778
-5.91	6272	-5.26189
-5.3	85185120	-5.16667
-5.334	69310	-5.12566
-5.547	6181	-5.10533
-5.222	10850	-5.02078
-5.555	4868	-4.96611
-5.406	11148	-4.87933
-4.857	1103	-4.57333

-4.995	2526	-4.53522
-4.645	424	-4.34156
-4.512	189087	-4.25122
-4.639	379	-4.23189
-4.48	13601	-4.20822
-4.494	1102	-4.20556
-4.503	11419	-4.20256
-4.372	13005	-4.15322
-4.679	614	-4.13734
-4.602	2762419	-3.99867
-4.51	13006	-3.98967
-4.107	564	-3.96667
-4.143	119	-3.95533
-4.153	13584	-3.911
-4.238	401	-3.90789
-4.307	12756	-3.90378
-4.473	12587	-3.89522
-4.179	7341	-3.88344
-4.188	31289	-3.87722
-4.33	11527	-3.87367
-4.107	10932	-3.873
-4.276	11266	-3.87211
-4.165	617	-3.851
-4.333	6915	-3.85033
-4.211	10930	-3.80389
-4.111	64956	-3.78678
-4.037	68352	-3.78122
-4.195	10430	-3.77533
-4.234	6657	-3.768
-4.345	10953	-3.75789
-4.456	17288	-3.75255
-4.089	186078	-3.73389
-4.248	10931	-3.72822
-3.9	8998	-3.72222
-4.243	20449	-3.71066
-4.324	10976	-3.67156
-4.181	58	-3.66167
-3.9	8174	-3.65556
-4.163	11559	-3.655
-4.031	602	-3.64378
-4.146	61138	-3.62989

-4.125	7360	-3.62989
-3.952	26331	-3.61378
-3.8	867	-3.61111
-4.234	12296	-3.60844
-3.9	8091	-3.6
-4.092	5281167	-3.58211
-3.876	441	-3.57433
-3.8	7524	-3.55556
-4.101	225710	-3.55233
-3.754	1045	-3.54456
-3.821	107712	-3.53656
-4.083	612	-3.53645
-3.8	9934	-3.53333
-3.977	262	-3.526
-3.883	594	-3.50967
-3.631	11747	-3.50411
-4.247	11173	-3.49311
-3.853	5281168	-3.45878
-3.6	779	-3.44444
-3.809	11428	-3.44422
-3.883	1060	-3.44244
-3.783	12041	-3.44033
-3.649	18522	-3.43178
-3.656	187	-3.43067
-3.687	239	-3.42867
-3.683	18467	-3.41
-3.7	7755	-3.4
-3.541	670	-3.38922
-3.6	824	-3.38889
-3.6	8871	-3.38889
-3.968	10111	-3.38378
-3.716	12301	-3.38056
-3.773	10351	-3.369
-3.6	8868	-3.34444
-3.743	1088	-3.33711
-3.827	5641	-3.33145
-3.498	563	-3.31467
-3.538	2723631	-3.31433
-3.781	286	-3.30789
-3.55	10442	-3.29356
-3.6	957	-3.28889

-3.608	673	-3.28233
-3.4	7757	-3.25555
-3.852	10341	-3.23489
-3.55	11126	-3.18089
-3.504	12251	-3.169
-3.3	753	-3.15556
-3.6	1030	-3.15555
-3.837	3520	-3.15155
-3.4	75606	-3.14445
-3.617	4	-3.12233
-3.4	138	-3.12222
-3.3	8103	-3.12222
-3.605	11613	-3.10745
-3.7	1032	-3.10544
-3.6	7971	-3.08889
-3.308	16590	-3.02556
-3.3	7976	-3.01111
-3.3	9023	-3
-3.4	971	-2.97778
-3.489	1048	-2.97211
-3.3	751	-2.92222
-3.646	1176	-2.89878
-3.2	7896	-2.88889
-3.2	82140	-2.88889
-3.32	178	-2.86578
-3.162	700	-2.83489
-3.104	1647	-2.83389
-3.1	750	-2.83333
-3.308	2365	-2.76211
-2.9	757	-2.73333
-3.189	10451	-2.71544
-2.9	9260	-2.66667
-3.11	11446286	-2.65589
-2.998	16592	-2.62845
-2.9	8454	-2.61111
-2.9	760	-2.58889
-2.655	6213	-2.53322
-2.905	66282	-2.47722
-2.8	8299	-2.41111
-2.543	1145	-2.31344
-2.659	6058	-2.26533

-2.5	795	-2.25555
-2.696	713	-2.18567
-2.4	767	-2.11111
-2.346	19310	-2.07222

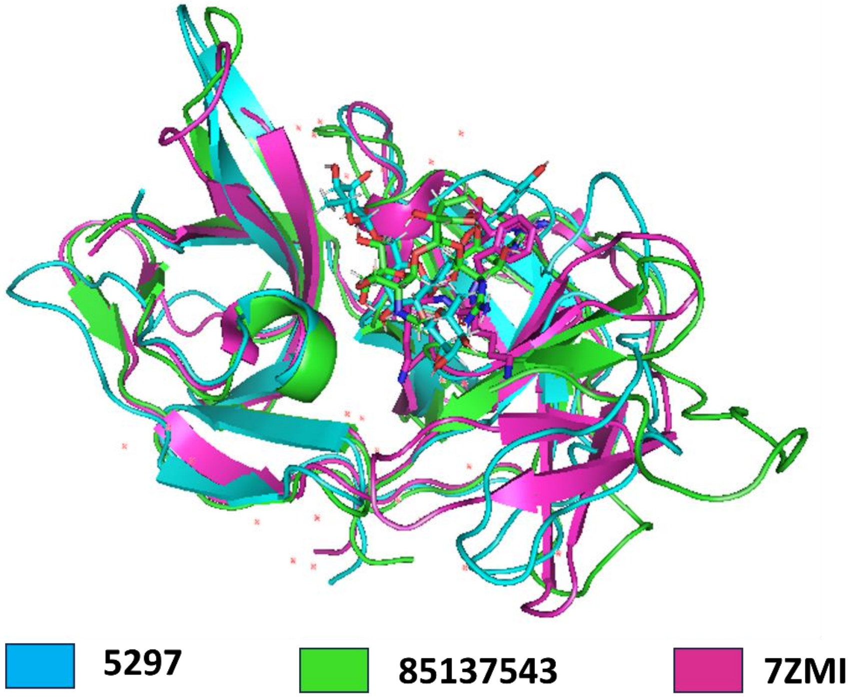

Figure S2. Structure of the protein-ligand complex of 5297 and 85137543 aligned over the crystal structure of 7ZMI.
